# “Boiled” Linseed Oil

**Published:** 1899-05

**Authors:** 


### THERAPEUTICAL AND PHARMACEUTICAL NOTES.

Under the Direction of W. J. Martin, M.D.C.,
KANKAKEE, ILL.

"Boiled" Linseed Oil. In the so-called boiling of linseed oil materials which are distinctly poisonous are added to increase the drying of the oil. Several of the oxides of lead have been and are probably still used for the purpose indicated, and compounds of cobalt, manganese, etc., have been recommended for the same use. Veterinarians should be very careful to ascertain before administering linseed oil that the oil be in the raw state and of undoubted purity. A case came under the writer's notice some time since, in which a horse-owner, unaware of the difference between the so-called "boiled" and raw oil, gave his horse a quart of the former, which undoubtedly caused the death of the animal.

